# Members of the Arabidopsis *FORKED1-LIKE* gene family act to localize PIN1 in developing veins

**DOI:** 10.1093/jxb/ery248

**Published:** 2018-07-03

**Authors:** Neema Prabhakaran Mariyamma, Kurtis J Clarke, Houlin Yu, Emily E Wilton, Jordan Van Dyk, Hongwei Hou, Elizabeth A Schultz

**Affiliations:** Department of Biological Sciences, University of Lethbridge, Lethbridge, AB, Canada

**Keywords:** Auxin canalization, DUF828, FORKED1, PIN1 localization, Pleckstrin-like homology domain, secretory pathway, vascular differentiation, vein pattern

## Abstract

The reticulate leaf vein pattern typical of angiosperms is proposed to have been a driving force for their evolutionary success. Vein pattern is established through auxin canalization via the auxin efflux protein PINFORMED1 (PIN1). During formation of vein loops, PIN1 cellular localization is increasingly restricted to either the basal side of cells in the lower domain or to the apical side in the upper domain. We previously identified the gene *FORKED1* (*FKD1*) to be required for PIN1 asymmetric localization and for the formation of closed vein loops. *FKD1* encodes a plant-specific protein with a domain of unknown function (DUF828) and a Pleckstrin-like homology domain. The Arabidopsis genome encodes eight similar proteins, which we term the *FORKED1-LIKE* (*FL*) gene family. Five FL family members localize primarily to the *trans*-Golgi network or the Golgi, and several co-localize with FKD1–green flourescent protein (GFP) and RABA1c, suggesting action in the secretory pathway. While single *FL* gene family mutations do not result in vein pattern defects, triple mutants with mutations in *FKD1*, *FL2*, and *FL3* result in a more symmetric PIN1 localization and a highly disconnected vein pattern. Our data suggest that *FL* genes act redundantly with *FKD1* in the secretory pathway to establish appropriate PIN1 localization in provascular tissue.

## Teaser

DUF828-containing proteins are unique to plants but, aside from FORKED1, their function is unknown. We find that DUF828 proteins act with FORKED1 to establish the reticulate leaf vein pattern.

## Introduction

The acquisition of characteristics imparting an increasingly complex vascular system represent key stages in plant evolution, allowing plants to be more successful in a terrestrial environment. The transition from a simple, dichotomously branching system to a reticulate pattern is thought to have been a major driving force behind angiosperm success. The vein hierarchies and vein meeting provide systems that are more efficient and better able to withstand both abiotic and biotic stresses (Roth-Nebelsick *et al.*, 2001; Brodribb and Feild, 2010; Feild *et al.*, 2011). Also, changes to leaf shape and size are correlated with changes to leaf vein pattern and density, suggesting co-ordination between the two processes (Dengler and Kang, 2001; Pahari *et al.*, 2014).

Vein pattern is established by directed auxin transport facilitated by the PINFORMED (PIN) proteins (Scarpella *et al.*, 2006; Wenzel *et al.*, 2007). The position of the midvein and secondary veins is defined through high levels of auxin response that are sequentially established within the leaf epidermis at the leaf margin. PIN1 proteins are localized within epidermal cells such that auxin is transported towards sequential convergence points that predict secondary veins. At these convergence points, auxin enters underlying cells, where it induces PIN transcription, establishing a PIN1 expression domain (PED) (Scarpella *et al.*, 2006). Subsequent events establish a group of cells that express basally localized PIN1 allowing auxin transport towards the midvein. During the process, PIN1 orientation changes within the PED, so that auxin moves from the peripheral cells into the central cell file; this process of canalization establishes a cell file through which auxin is transported and which predicts the position of the lower loop domain (LLD) of the secondary vein (Scarpella *et al.*, 2006; Wenzel *et al.*, 2007). Subsequently, PIN1 expression is induced in a group of cells above the lower loop; these cells predict the upper loop of the secondary vein (upper loop domain, ULD). PIN1 is initially localized basally within cells of the ULD, but apical localization within cells of the upper regions allows auxin flux into the upper midvein, establishing a closed loop (Scarpella *et al.*, 2006; Hou *et al.*, 2010). Induction of tertiary and quaternary veins seems not to depend on margin convergence points, but rather may occur through auxin synthesis within the developing lamina (Aloni *et al.*, 2003; Cheng *et al.*, 2006, 2007).

The spacing of secondary veins is dependent on the spacing of the auxin convergence points on the leaf margin epidermis. In the absence of *UNHINGED* (*UNH*), which is a part of the Golgi-associated retrograde protein complex required for vacuole function, fewer convergence points are established and fewer secondary veins are made (Pahari *et al.*, 2014). The meeting of veins is dependent on PIN1 localization to the apical side of cells within the ULD. In *FORKED1* (*FKD1*) mutants, PIN1 fails to localize to the apical side, and veins do not meet distally (Steynen and Schultz, 2003; Hou *et al.*, 2010). FKD1 forms a complex with SCARFACE (SFC) (Naramoto *et al.*, 2009), and both proteins are localized to the *trans*-Golgi network (TGN) and to vesicles defined by RABA-GTPases (Prabhakaran Mariyamma *et al.*, 2017). Thus, we propose that FKD1 acts in a secretory process that targets PIN1 to the apical cell membrane.

The FKD1 protein contains a domain of unknown function828 (DUF828) and a plant Pleckstrin homology-like (PH_2) domain (Hou *et al.*, 2010). The plant-specific DUF828 domain is found in eight other proteins within the Arabidopsis genome; we define this group of genes as the *FORKED1-LIKE* (*FL*) gene family. In this study, we used a combination of cellular localization of proteins and mutant analysis to establish *FL* gene function. Through these approaches, we propose that several of the FL proteins are localized to the TGN and secretory pathway where they act redundantly with FKD1 to localize PIN1 in developing veins, establishing a reticulate vein pattern.

## Materials and methods

### Identifying DUF828 domain evolution in the plant kingdom

In Arabidopsis, nine genes are annotated as encoding DUF828 domains within the Pfam30 database (Finn *et al.*, 2016). On the basis of their similarity to the *FKD1* gene (Hou *et al.*, 2010), the eight genes were termed the Arabidopsis *FORKED1-LIKE* (*FL*) genes and were named *FKD1-LIKE* (*FL*) *1–8: FL1*=At5g43870, *FL2*=At3g22810, *FL3*=At4g14740, *FL4*=AT4g32780, *FL5*=At4g17350, *FL6*=At5g47440, *FL7*=At4g16670, and *FL8*=At5g57770 (Supplementary Table S1 at *JXB* online). Using Clustal Omega (Sievers *et al.*, 2011), we aligned protein sequences identified within the Pfam database as containing the DUF828 domain from Arabidopsis and other representative species including: *Amborella trichopoda*1 (evm_27.model.AmTr_v1.0_scaffold00019.130_AMBTC), *A. trichopoda*2 (evm_27.model.AmTr_v1.0_scaffold00176.33_AMBTC), *A. trichopoda*3 (evm_27.model.AmTr_v1.0_scaffold00109.149_AMBTC), *A. trichopoda* 4 (evm_27.model.AmTr_v1.0_scaffold0002.606_AMBTC), *Marchantia polymorpha* (AXG93_4027s1220), *Oryza sativa* Os02g44040.1 (Os02g44040.1_ORYSA), *O. sativa* Os10g41060.1 (Os10g41060.1_ORYSA), *O. sativa* Os01g13070.1 (Os01g13070.1_ORYSA), *O. sativa* Os12g41140.1 (Os12g41140.1_ORYSA), *O. sativa* Os03g43510.1 (Os03g43510.1_ORYSA), *O. sativa* Os10g41870.1 (Os10g41870.1_ORYSA), *P. abies* 10432316g0010 (MA_10432316g0010_ PICAB), *Picea abies* 340269g0010 (MA_ 340269g0010_PICAB), *P. abies* 39658g0010 (MA_39658g0010_PICAB), *P. abies* 0368g0010 (MA_90368g0010_PICAB), *P. patens* Pp1s155_109V6.1 (Pp1s155_109V6.1_PHYPA), *Selaginella moellendorffii* 269293 (selmo_269293_SELMO), *S. moellendorffii* 268691 (selmo_268691_SELMO), *S. moellendorffii* 267397 (selmo_267397,_SELMO), and *S. moellendorffii* 443866 (selmo_443866_SELMO). The aligned sequences were used to construct a phylogenetic tree with *M. polymorpha* as the outgroup, utilizing MEGA7 (Kumar *et al.*, 2016), a maximum-likelihood tree reconstruction method employing the Neighbor–Joining tree algorithm (500 replications with default parameters). A second phylogenetic tree was created using the nine sequences from *Arabidopsis thaliana,* using one of the *S. moellendorffii* proteins as an outgroup.

### Plant material and characterization of alleles

*Nicotiana tabacum* seeds were obtained from Michigan State University, USA. SYP61pro:SYP61:CFP (cyan fluorescent protein) seeds were obtained from Dr Marissa Utegui, University of Wisconsin, USA. PIN1–GFP (green fluorescent protein) seeds and SALK T-DNA insertion lines (Alonso *et al.*, 2003) for the members of the *FL* gene family (*fl1-1*=Salk 124321; *fl1-2*=Salk 064024, *fl2*=Salk 026656; *fl3*=Salk 013371; *fl6*=Salk 063367; *fl7*=Salk 077717) were obtained from the Arabidopsis Biological Resource Centre at Ohio State University, USA. Plants homozygous for insertions were identified by PCR using gene-specific primers (Supplemental Table S2), and the genomic region adjacent to the T-DNA left border was sequenced following amplification by PCR. To assess transcript levels within seedlings homozygous for the insertion alleles, 100 mg of wild-type, *fl1-1*, *fl1-2*, *fl2*, *fl3*, *fl6*, and *fl7* Arabidopsis seedlings at 6 days after germination (DAG) were frozen in liquid nitrogen, and RNA was extracted using Trizol™ (Ambion) according to the manufacturer’s protocol. Following determination of RNA quality and concentration by TAE gel electrophoresis and spectrophotometry, 1 μg was reverse transcribed using oligo(dT) primers according to the manufacturer’s protocol (Easyscript; ABM). The resulting cDNA provided a template for RT-PCR using *FL* gene-specific primers or primers to the gene *PP2A* (Czechowski *et al.*, 2005) for either 30 or 35 cycles under the following conditions (Supplementary Table S2): initial denaturation (94 °C for 2 min), amplification (94 °C for 30 s, annealing for 30 s, 72 °C for 30 s), and final elongation (72 °C for 5 min). RT-PCR products were observed following separation on a 1.5% sodium borate gel.

### Growth conditions of Arabidopsis and *Nicotiana tabacum*

As described previously (Pahari *et al.*, 2014), Arabidopsis was sown on soil or plates with AT growth medium (Ruegger *et al.*, 1998), left at 4 °C for 3 d, and transferred to the growth chamber. The date of transfer was considered 0 DAG. The Arabidopsis Columbia (Col-0) ecotype was used as a wild-type control in all experiments. Plants were grown in growth chambers at 60% humidity, 22 °C, and continuous light intensity of ~130 μmol photons m^−2^ s^−1^ from Sylvania Cool White, Grow Lux and 60 W frosted incandescent bulbs (Osram Sylvania Inc, Danvers, MA, USA). *Nicotiana tabacum* seeds were sown on soil and treated in the same way as the Arabidopsis until 14 DAG, after which seedlings were transplanted into individual pots and grown under 16 h of light at 22 °C and 8 h of dark at 18 °C in 60% relative humidity. Approximately 4 weeks after germination, plants were injected with *Agrobacterium* for transient gene expression.

### Generation of transgenes for transient or stable expression

Vectors containing full-length cDNA (Yamada *et al.*, 2003) for members of the *FL* gene family, U83719 (*FL1*), U19780 (*FL3*), S67212 (*FL5*), S67215 (*FL6*), S69284 (*FL7*), and the pnigel07 vector (Geldner *et al.*, 2009), were obtained from ABRC. 35S:ST-RFP (red fluorescent protein) (Renna *et al.*, 2005) was obtained from Dr Federica Brandizzi, Michigan State University, USA, 35S:GFP–RABA1c (Qi and Zheng, 2013) from Dr Hugo Zheng, McGill University, Canada, and 35S:mRFP–SYP61 (Choi *et al.*, 2013) from Dr Takashi Ueda, University of Tokyo, Japan.

Generation of the 35S:FKD1–GFP construct was described previously (Prabhakaran Mariyamma *et al.*, 2017). Generation of constructs for transient expression in *N. tabacum* was done by recombining cDNAs from the *FL* gene families with the pnigel07 [yellow fluorescent potein (YFP)] vector using the Cre/lox system as previously described (Geldner *et al.*, 2009). The pnigel07 vector places the fusion protein under the control of the pUBQ10 promoter, generating vectors pUBQ10:FL1-YFP, pUBQ10:FL3-YFP, pUBQ10:FL5-YFP, pUBQ10:FL6-YFP, and pUBQ10:FL7-YFP. Vectors were sequenced to confirm fidelity using previously described primers (Geldner *et al.*, 2009).

pUBQ10:FL3-YFP was transformed via *Agrobacterium tumefaciens* into the wild type (Col-0) using the floral dip method (Clough and Bent, 1998). Transformed plants were selected for Basta resistance. For transient expression in *N. tabacum*, the abaxial leaf epidermis was injected with *Agrobacterium* strains harbouring appropriate binary vectors following a previous protocol (Batoko *et al.*, 2000).

### Generation of multiple mutant lines between *FKD1* and members of the *FL* gene family

Homozygous T-DNA insertions in *FL1*, *FL2*, *FL3*, *FL6*, and *FL7* did not show any obvious leaf vein phenotype. Hence, multiple mutant lines were created between *FKD1* and various members of the *FL* gene family. Plants homozygous for insertions in *FL1*, *FL2*, *FL3*, *FL6*, and *FL7* were crossed with *fkd1*. Homozygous double mutants of *fkd1*/*fl1-1*, *fkd1*/*fl1-2, fkd1*/*fl2*, and *fkd1*/*fl3* were identified by screening plants with the *fkd1* phenotype by PCR to confirm the T-DNA insertion using specific primer combinations. To generate the *fkd1*/*fl2/fl3* triple mutants, double mutants of *fkd1*/*fl2* and *fkd1*/*fl3* were crossed and triple mutants identified in the F_2_ generation. To generate the *fkd1/fl6/fl7* triple mutants, plants heterozygous for *fkd1*/*fl6* and *fkd1/fl7* were crossed and plants homozygous for *fkd1* and both insertions were identified in the F_2_ generation. To generate the *fkd1/fl1-1*/*fl2/fl3* and *fkd1/fl1-2*/*fl2/fl3* quadruple mutant, the *fkd1*/*fl2/fl3* triple mutant was crossed with double mutant *fkd1*/*fl1-1* or *fkd1/fl1-2*, and quadruple mutants were identified in the F_3_ generations.

A stable transgenic line of the triple mutant expressing PIN1–GFP was generated by crossing plants homozygous for *fkd1* and PIN1–GFP with the *fkd1/fl2/fl3* triple mutant line. The F_1_ generation was backcrossed to *fkd1/fl2/fl3* and a homozygous line was identified in the F_2_ generation by screening for *fkd1* phenotype, confirming the presence of T-DNA insertions by PCR and checking for PIN1–GFP expression.

### Confocal imaging and analysis

For transient expression analysis, pieces of *N. tabacum* leaves 48 h post-injection were mounted in water. For stable expression, Arabidopsis roots or cotyledons at 2.5 DAG or leaves at sequential days were mounted in water. Tissue was viewed under ×40 or ×60 magnification oil-immersion objectives with an Olympus Fluoview FV1000 confocal microscope. For co-localization experiments involving YFP and RFP, YFP and RFP were excited with laser 473 nm (emission filters 485–545 nm) and 559 nm (emission filters 570–670 nm), respectively. For co-localization experiments involving GFP with YFP, GFP was excited with laser 458 nm (emission filters 470–496 nm) and YFP with the 515 nm (emission filters 530–600 nm). For co-localization experiments involving YFP and CFP, YFP and CFP were excited sequentially with 405 nm and 515 nm lasers, respectively. Imaging was carried out using the line-sequential scanning mode, and all images used in comparisons were taken at the same confocal settings. To assess the co-localization of proteins, Pearson’s coefficient of correlation (PCC) values were obtained within single cells (cotyledons and leaves) using the PSC co-localization plugin in NIH Image J software (French *et al.*, 2008; Schneider *et al.*, 2012). To determine the frequency with which FL-labelled punctae are also labelled by various other markers, the number of punctae labelled with both markers was divided by the number of punctae labelled by only the FL family member. For both co-localization and frequency analyses, at least 20 samples were analysed for each experiment. Images shown in the figures are representative of average co-localization patterns for the samples. Images were processed with Adobe Photoshop Elements version 5.0 software (Adobe Systems).

### Morphological characterization of T-DNA insertion mutants

For analysis of cotyledon and leaf area, vein density, vein meeting, and vascular islands (VIs), cotyledons and first leaves were taken from various genotypes at 14 and 21 DAG, respectively, decolorized overnight in 70% ethanol, followed by clearing in chloral hydrate (8 chloral hydrate:2 glycerol:1 water) for 1 week. When clear, they were mounted in 66% glycerol and images were taken using a Nikon Cool Pix 990 camera mounted on a Leica MZ8 microscope. Image J software was used to measure cotyledon and leaf characteristics. Area and vein length were obtained by tracing the whole cotyledon/leaf area and all veins, and vein density was calculated by dividing the total length by area.

For analysis of root elongation and gravitropism, seedlings of the wild type, *fkd1*, *fkd1/fl2/fl3*, and *fkd1*/*fl1-2/fl2/fl3* were grown vertically on Petri plates with AT medium at a density of 10 plants per plate. Primary root length measurements were performed by photographing roots at 4 DAG and, 24 h later, merging images and measuring the root growth between the two images in Image J. Analysis of root gravitropism was done by rotating vertically growing 5 DAG seedlings by 90°. Images of the root tips before and 6 h after rotation were captured, and the change in the angle of root tip was measured by using Image J.

To analyse shoot characteristics, genotypes were grown on soil as described above. Flowering time was defined as petal opening of the first flower, at which time the number of rosette leaves and bracts was counted.

### Statistical analysis

An ANOVA was carried out for all analyses with a sample size of *n* >30 using the PROC MIXED procedure of SAS. Means were then compared using the least squares mean linear hypothesis test (LSMEANS/PDIFF). Treatment effects were declared significant at *P*<0.05. For sample size (*n* <30) Student’s *t*-test was conducted to determine if the compared samples were significantly different from each other (*P*<0.05). Fisher’s test was carried out to determine the statistical difference between the genotypes for measurements with a mean value of zero (VIs, frequency of non-meeting veins, FKD1–GFP expression in mutant lines, and PIN1 localization in developing veins).

## Results

### Expansion of the DUF828-containing family within the plant kingdom

The *FKD1* gene encodes a protein with a plant-specific ‘domain of unknown function’ [DUF828; InterPro (IPR) domain IPR008546] and a plant-specific Pleckstrin homology-like domain (PH_2; IPR013666) (Hou *et al.*, 2010). Comparison of domain structure amongst those proteins identified as having a DUF828 in the Pfam30 database (Finn *et al.*, 2016) indicates that DUF828 is often associated with either a PH_2 or a Pleckstrin homology (PH) domain. DUF828 together with PH_2 appears in bryophytes, with a single copy in the liverwort *Marchantia polymorpha* and the moss *Physcomitrella patens*. The co-occurrence of the two domains in bryophytes suggests that their association is the ancestral state, with subsequent losses a derived condition. The presence of the genes in non-vascular plants indicates that they have a function that pre-dates vascular cells. The lycophyte *Selaginella moellendorffii*, the gymnosperm *Picea abies*, and the basal angiosperm *Amborella trichopoda* each possess four copies of DUF828 genes. Among derived angiosperms, eudicots and monocots have from 5 (e.g. *Vitis vinifera*) to 16 copies (e.g. *Glycine max*) of the genes. The conservation and expansion of the DUF828 family over 400 million years of terrestrial plant evolution suggests that it is involved in key biological functions.

**Fig. 1.**
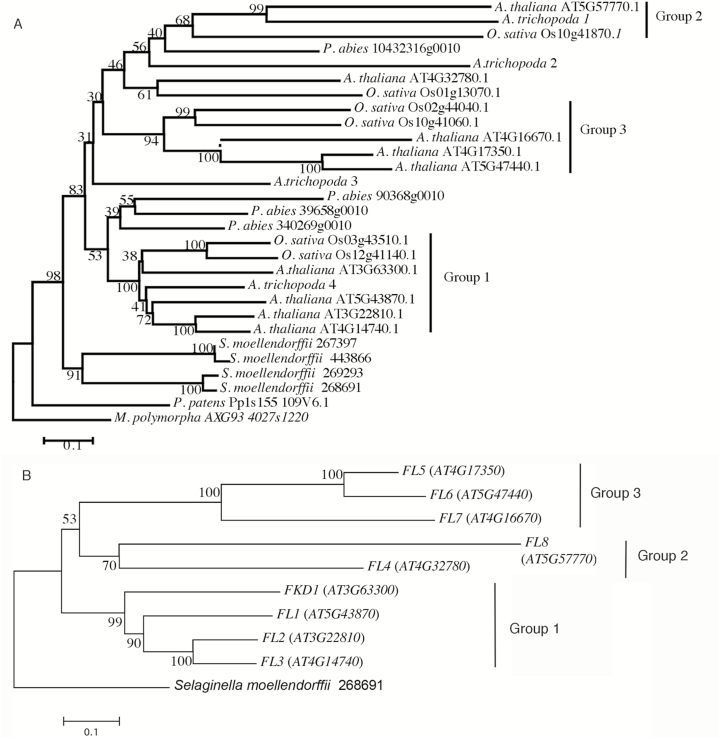
Phylogenetic tree of (A) DUF828 domain sequences from diverse species in the plant kingdom and (B) DUF828-encoding genes (*FL* gene family) in *Arabidopsis thaliana.* Phylogenetic tree of proteins from representative species *Marchantia polymorpha* subsp *Polymorpha*, *Physcomitrella patens*, *Selaginella moellendorffii*, *Picea abies*, *Amborella trichopoda*, *Oryza sativa*, and *Arabidopsis thaliana*. Bootstrap values over 500 replicates are given at each branch. Strongly supported Angiosperm groups (bootstrap value >75) are indicated as groups 1, 2, and 3. (B) Phylogenetic tree of *FL* gene family (At3g63300, *FKD1*; At5g43870, *FL1*; At3g22810, *FL2*; At4g14740, *FL3*; At4g32780, *FL4*; At4g17350, *FL5*; At5g474400, *FL6*; At4g16670, *FL7*, and At5g57770, *FL8*) in *A. thaliana.* Groups 1, 2, and 3 correspond to those in (A).

To assess the relatedness amongst members of the protein family, we undertook a phylogenetic analysis using MEGA7 (Kumar *et al.*, 2016). A phylogenetic tree using *M. polymorpha* as the outgroup was created with proteins from species representative of key groups including *P. patens*, *S. moellendorffii*, *P. abies*, *A. trichopoda*, *O. sativa*, and *A. thaliana* (Fig. 1A). The tree places the four *S. moellendorffii* proteins in a single clade basal to the gymnosperm and angiosperm sequences, indicating that the last common vascular plant ancestor had only one gene copy and that the *S. moellendorffii* proteins result from recent duplication events. Resolution amongst the gymnosperm and angiosperm sequences is poor, forming two clades with weak support (bootstrap value 53 over 500 replicates). *Picea abies* proteins are present in both clades, which might indicate that the last common ancestor had two gene copies. Three clades (groups 1, 2, and 3) of angiosperm proteins are strongly supported (bootstrap values 100, 68, and 94, respectively). Both groups 1 and 2 contain an *A. trichopoda* protein, suggesting that the ancestral angiosperm contained at least two copies of the *FL* gene family.

Nine Arabidopsis proteins, including FKD1, are annotated as containing DUF828. We have named these genes the Arabidopsis *FORKED1-LIKE* (*FL*) family, and the eight members in addition to *FKD1* were named *FL1–FL8*. The biological function of none of these proteins is known. To better see the relationships amongst the FL family proteins, we generated a phylogenetic tree of the Arabidopsis FL proteins using one of the *S. moellendorffii* proteins as an outgroup (Fig. 1B). The FL family forms three strongly supported clades, group 1 (FKD1 and FL1–FL3), group 2 (FL4 and FL8), and group 3 (FL5–FL7). Within group 1, FKD1 is the most divergent, which may indicate less functional overlap with other members of the gene family and may explain the visible phenotype when *FKD1* alone is defective. Sequence alignment using ClustalW reveals that the other members of group 1 are >50% similar to FKD1 over the entire sequence, while similarity of proteins within the other two clades (FL4–FL8) to FKD1 is much lower (Supplementary Table S1).

### Divergent cellular localization of the FL family members

The cellular function of none of the *FL* genes has been defined. As a first step towards understanding their function at the cellular level, we sought to determine the cellular localization of the FL family proteins by generating YFP fusions of several FL family members: two members of group 1 (pUBQ10:FL1-YFP and pUBQ10:FL3-YFP) and three members of group 3 (pUBQ10:FL5-YFP, pUBQ10:FL6-YFP, and pUBQ10:FL7-YFP), and transformed them into *N. tabacum* and wild-type Arabidopsis (pUBQ10:FL3-YFP). Like FKD1 (Prabhakaran Mariyamma *et al.*, 2017), all the members of the FL family label punctae of variable sizes, some of which are motile.

**Fig. 2.**
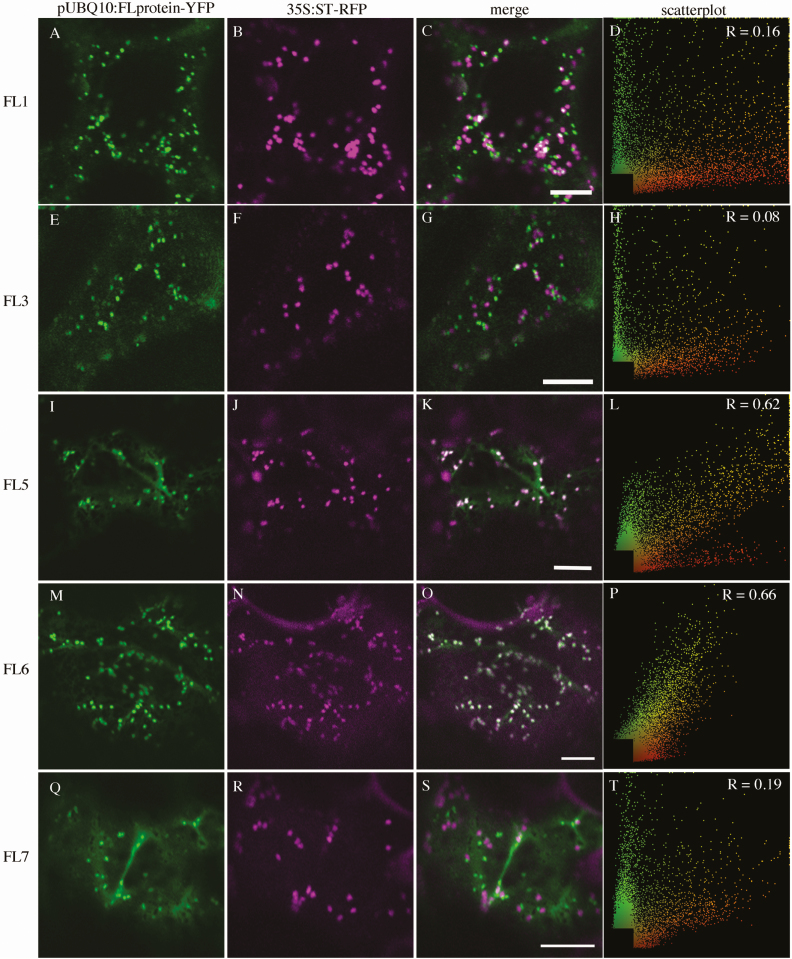
Subcellular localization of FL family members with 35S:ST–RFP transiently expressed in *Nicotiana tabacum*. (A), (E), (I), (M), and (Q) are pUBQ10:FL-YFP alone; (B), (F), (J), (N), and (R) are 35S:ST–RFP alone; (C), (G), (K), (O), and (S) are the merged images. (D), (H), (L), (P), and (T) are scatterplots of the merged image with Pearson’s coefficient of correlation (*R*) values (scale bar=10 μm).

**Fig. 3.**
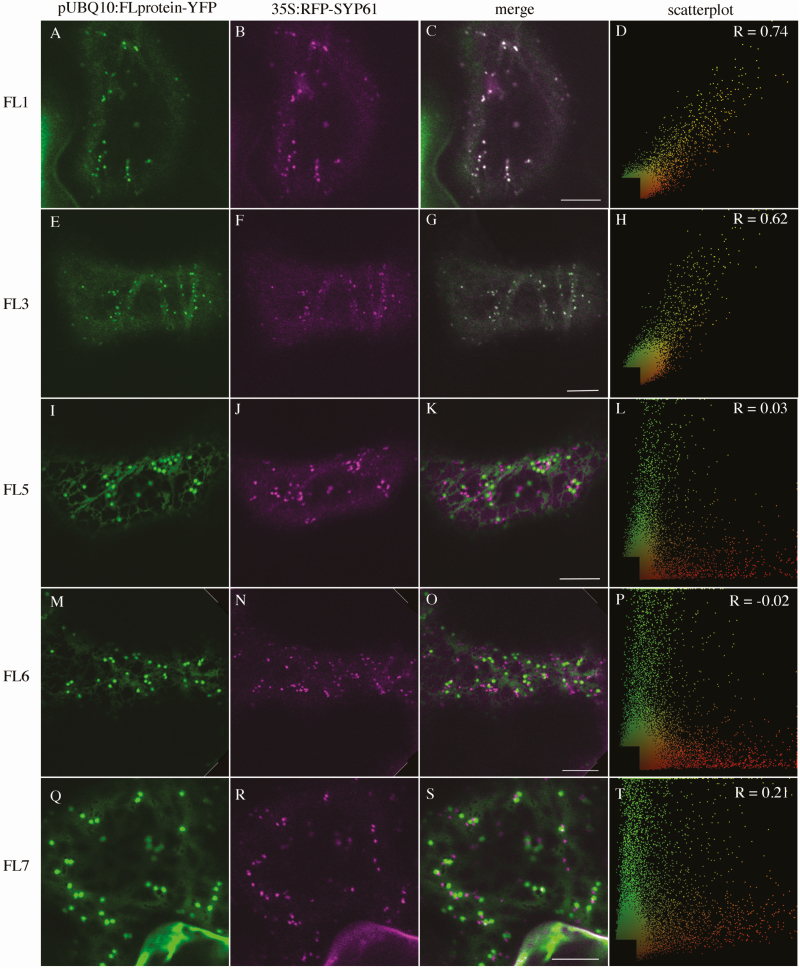
Subcellular localization of *FL* gene family members with 35S:mRFP–SYP61 transiently expressed in *Nicotiana tabacum*. (A), (E), (I), (M), and (Q) are pUBQ10:FL-YFP alone; (B), (F), (J), (N), and (R) are 35S:mRFP–SYP61 alone; (C), (G), (K), (O), and (S) are the merged images. (D), (H), (L), (P), and (T) are scatter plots of the merged image with Pearson’s coefficient of correlation (*R*) values (scale bar=10 μm).

We first performed co-localization experiments with 35S:ST–RFP, a marker of the Golgi apparatus (GA) (Boevink *et al.*, 2002) and with mRFP–SYP61, a marker of the TGN (Choi *et al.*, 2013). The two members of group 1 do not associate with 35S:ST–RFP (pUBQ10:FL1-YFP, PCC=0.09 ± 0.21; pUBQ10:FL3-YFP, PCC=0.05 ± 0.19, Table 1; Fig. 2A–H). Whereas two members of group 3 associate moderately with 35S:ST–RFP (pUBQ10:FL5-YFP, PCC=0.61 ± 0.20; pUBQ10:FL6-YFP, PCC=0.61 ± 0.16, Table 1; Fig. 2I–P), FL7, the third member of group 3, associates weakly (PCC=0.17 ± 0.15, Table 1; Fig. 2Q–T). Association with 35S:mRFP–SYP61 is opposite, with members of group 1 associating strongly (pUBQ10:FL1-YFP, PCC=0.69 ± 0.13; pUBQ10:FL3-YFP, PCC=0.63 ± 0.09, Table 1, Fig. 3A–H), and members of group 3 showing either no association (pUBQ10:FL5-YFP, PCC=0.05 ± 0.13; pUBQ10:FL6-YFP, PCC= –0.04 ± 0.11, Table 1; Fig. 3I–P) or weak association (pUBQ:FL7-YFP PCC=0.12 ± 0.22, Table 1; Fig. 3Q–T). In stably transformed Arabidopsis, pUBQ10:FL3-YFP also co-localizes moderately with SYP61–CFP (PCC=0.38 ± 0.12, Table 1). When we counted the proportion of punctae labelled by an FL–YFP fusion that were also labelled by either 35S:ST–RFP or 35S:mRFP–SYP61, we found that >95% of FL1–YFP- (0.96 ± 0.03) and FL3–YFP- (0.98 ± 0.02) labelled punctae were also labelled with 35S:mRFP–SYP61, whereas >90% of FL5–YFP- (0.91 ± 0.24) and FL5–YFP- (0.99 ± 0.01) labelled punctae were also labelled with 35S:ST–RFP. In contrast, <10% of FL7–YFP-labelled punctae were also labelled with either 35S:ST–RFP (0.08 ± 0.04) or 35S:mRFP–SYP61 (0.06 ± 0.06). The co-localization data suggest that the group 1 proteins are primarily localized to the TGN, whereas group 3 proteins are primarily localized to the GA, with the exception of FL7, which shows weak localization to both the TGN and GA.

**Table 1.** Correlation of expression between the intensities of FL family members fused to YFP and ST–RFP, SYP61–RFP, SYP61–CFP, FKD1–GFP, or 35S:GFP–RABA1c in leaf epidermis of *Nicotiana tabacum* and in the cotyledon epidermis of Arabidopsis seedlings at 2.5 DAG

FL family fusion	Marker	*n*	Tissue	Mean PCC	Frequency of FL family with marker
pUBQ10:FL1-YFP	35S:ST–RFP	28	*N. tabacum*	0.09 ± 0.21	0.13 ± 0.06
pUBQ10:FL3-YFP	35S:ST–RFP	25	*N. tabacum*	0.05 ± 0.19	0.11 ± 0.05
pUBQ10:FL5-YFP	35S:ST–RFP	25	*N. tabacum*	0.61 ± 0.20	0.91 ± 0.24
pUBQ10:FL6-YFP	35S:ST–RFP	22	*N. tabacum*	0.61 ± 0.16	0.99 ± 0.01
pUBQ10:FL7-YFP	35S:ST–RFP	21	*N. tabacum*	0.17 ± 0.15	0.08 ± 0.04
pUBQ10:FL1-YFP	35S:SYP61–RFP	30	*N. tabacum*	0.69 ± 0.13	0.96 ± 0.03
pUBQ10:FL3-YFP	35S:SYP61–RFP	25	*N. tabacum*	0.63 ± 0.09	0.98 ± 0.02
pUBQ10:FL3-YFP	SYP61pro:SYP61–CFP	54	*Arabidopsis*	0.38 ± 0.12	0.71 ± 0.11
pUBQ10:FL5-YFP	35S:SYP61–RFP	28	*N. tabacum*	0.05 ± 0.13	0.11 ± 0.11
pUBQ10:FL6-YFP	35S:SYP61–RFP	37	*N. tabacum*	–0.04 ± 0.11	0.06 ± 0.04
pUBQ10:FL7-YFP	35S:SYP61–RFP	24	*N. tabacum*	0.12 ± 0.22	0.06 ± 0.06
pUBQ10:FL1-YFP	35S:FKD1–GFP	25	*N. tabacum*	0.19 ± 0.25	0.22 ± 0.16
pUBQ10:FL3-YFP	35S:FKD1–GFP	44	*N. tabacum*	0.37 ± 0.25	0.26 ± 0.26
pUBQ10:FL3-YFP	35S:FKD1–GFP	25	*Arabidopsis*	0.46 ± 0.26	0.25 ± 0.15
pUBQ10:FL5-YFP	35S:FKD1–GFP	48	*N. tabacum*	0.20 ± 0.22	0.22 ± 0.20
pUBQ10:FL6-YFP	35S:FKD1–GFP	27	*N. tabacum*	–0.07 ± 0.22	0.05 ± 0.06
pUBQ10:FL7-YFP	35S:FKD1–GFP	46	*N. tabacum*	0.76 ± 0.21	0.87 ± 0.32
pUBQ10:FL1-YFP	35S:GFP–RABA1c	22	*N. tabacum*	0.76 ± 0.13	0.36 ± 0.09
pUBQ10:FL3-YFP	35S:GFP–RABA1c	35	*N. tabacum*	0.75 ± 0.12	0.60 ± 0.19
pUBQ10:FL5-YFP	35S:GFP–RABA1c	23	*N. tabacum*	0.34 ± 0.19	0.21 ± 0.28
pUBQ10:FL6-YFP	35S:GFP–RABA1c	23	*N. tabacum*	0.01 ± 0.12	0.21 ± 0.00
pUBQ10:FL7-YFP	35S:GFP–RABA1c	31	*N. tabacum*	0.68 ± 0.27	0.86 ± 0.29

Mean PCC was determined using the co-localization plugin in NIH image J from the intensity scatterplot of merged images. Frequency of FL family fusion with each marker was determined by counting the number of punctae labelled by both markers, and dividing it by the number of punctae labelled by the FL family protein

**Fig. 4.**
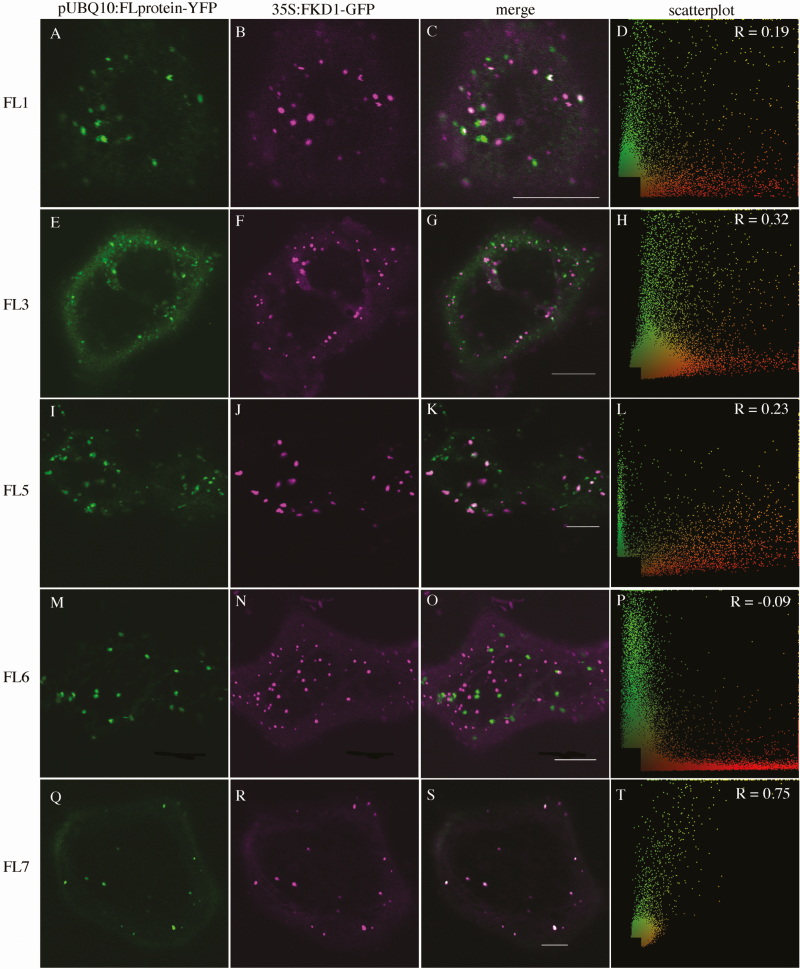
Subcellular localization of FL family members with 35S:FKD1–GFP transiently expressed in *Nicotiana tabacum*. (A), (E), (I), (M), and (Q) are pUBQ10:FL-YFP alone; (B), (F), (J), (N), and (R) are 35S:FKD1–GFP alone; (C), (G), (K), (O), and (S) are the merged images. (D), (H), (L), (P), and (T) are scatter plots of the merged image with Pearson’s coefficient of correlation (*R*) values (scale bar=10 μm).

To delineate further the cellular localization of the FL family proteins and to determine whether they might act redundantly with FKD1, we analysed their co-localization with 35S:FKD1–GFP (Table 1; Fig. 4). As might be expected from its strong localization to the TGN, pUBQ10:FL3-YFP co-localizes moderately with 35S:FKD1–GFP both in *N. tabacum* (PCC=0.37 ± 0.25, Table 1; Fig. 4E–H) and in stably transformed Arabidopsis (PCC=0.46 ± 0.26, Table 1). Surprisingly, despite its fairly strong localization to the TGN, pUBQ10:FL1-YFP only weakly associates with 35S:FKD1–GFP (PCC=0.19 ± 0.25, Table 1; Fig. 4A–D), whereas pUBQ10:FL7-YFP, which does not strongly associate with the TGN, co-localizes very strongly with 35S:FKD1–GFP (PCC=0.76 ± 0.21 Table 1; Fig. 4Q–T). Consistent with their stronger Golgi association, pUBQ10:FL5-YFP co-localizes weakly with 35S:FKD1–GFP (PCC=0.20 ± 0.22, Table 1; Fig. 4I–L) and pUBQ10:FL6-YFP does not co-localize with 35S:FKD1–GFP (PCC= –0.07 ± 0.22, Table 1; Fig. 4M–P). When we counted the proportion of punctae labelled by an FL–YFP fusion that were also labelled by 35S:FKD1–GFP (Table 1), we found that >20% of FL1–YFP- (0.22 ± 0.16), FL3–YFP- (0.26 ± 0.26), or FL5–YFP- (0.22 ± 0.20), and >85% of FL7–YFP- (0.87 ± 0.32) labelled punctae were also labelled with FKD1–GFP, while very few punctae labelled with FL6–YFP were also labelled by FKD1–GFP (0.05 ± 0.06). While the varying localization of the FL proteins suggests that they probably have distinct roles within the endomembrane pathway, their weak to moderate localization with FKD1 does not exclude the possibility that the proteins act in a similar pathway and may function redundantly.

**Fig. 5.**
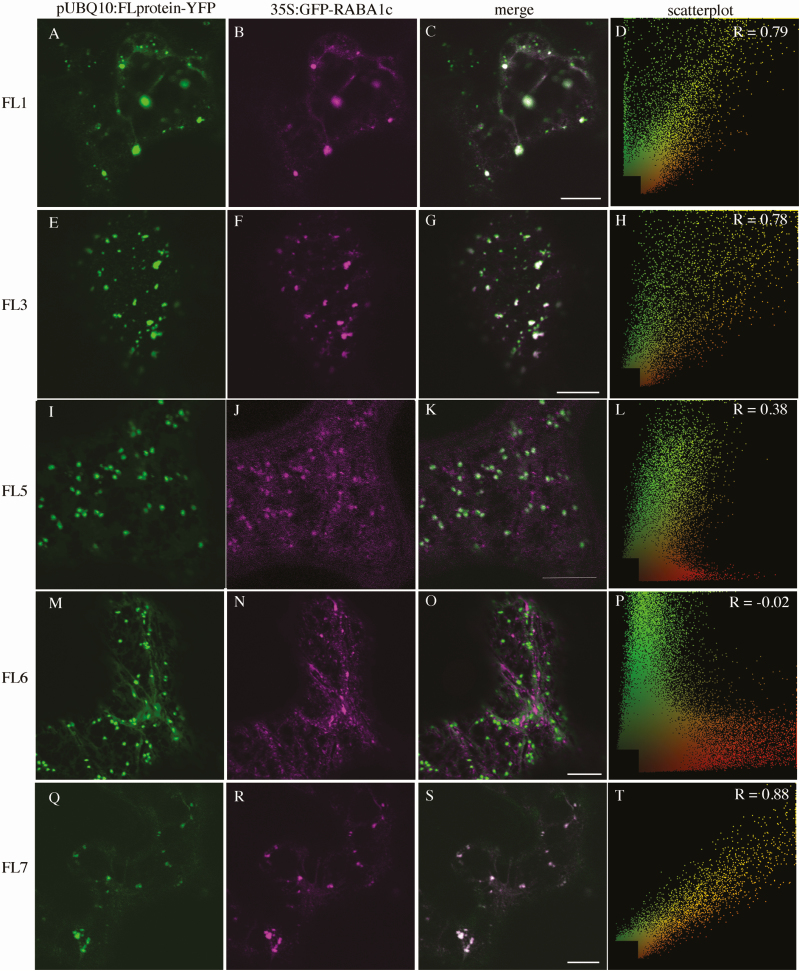
Subcellular localization of FL family members with 35S:GFP–RABA1c transiently expressed in *Nicotiana tabacum*. (A), (E), (I), (M), and (Q) are pUBQ10:FL-YFP alone; (B), (F), (J), (N), and (R) are 35S:GFP–RABA1c alone; (C), (G), (K), (O), and (S) are the merged images. (D), (H), (L), (P), and (T) are scatter plots of the merged image with Pearson’s coefficient of correlation (*R*) values (scale bar=10 μm).

We have recently shown that FKD1 co-localizes with members of the RABA family, suggesting a role in secretion (Prabhakaran Mariyamma *et al.*, 2017). To test if other members of the FL family also localize to a secretory compartment, we assessed their co-localization with 35S:GFP–RABA1c, which co-localizes strongly to the TGN (VTI12–YFP) and moderately to the *trans*-Golgi (ST–YFP) (Qi and Zheng, 2013). We found that, when transiently expressed in *Nicotiana*, pUBQ10:FL1-YFP, pUBQ10:FL3-YFP, or pUBQ10:FL7-YFP co-localized strongly with 35S:GFP–RABA1c (PCC=0.76 ± 0.13; 0.75 ± 0.12, and 0.68 ± 0.27 respectively, Table 1; Fig. 5A–H, Q–T), whereas pUBQ10:FL5-YFP co-localized moderately with 35S:GFP–RABA1c (PCC=0.34 ± 0.19, Table 1; Fig. 5I–L) and pUBQ10:FL6-YFP did not co-localize with 35S:GFP–RABA1c (PCC=0.01 ± 0.12, Table 1; Fig. 5M–P). When we counted the proportion of punctae labelled by an FL–YFP fusion that were also labelled by 35S:GFP–RABA1c, we found a similar trend, but with some key differences possibly because the fairly strong cytosolic localization of 35S:GFP–RABA1c affected the PCC values. More than one-third of FL1–YFP- (0.36 ± 0.09), more than one-half of FL3–YFP- (0.60 ± 0.19), and >85% of FL7–YFP- (0.87 ± 0.32) labelled punctae were also labelled with 35S:GFP–RABA1c, whereas less than a quarter of FL5–YFP (0.21 ± 0.28) and no FL6–YFP (0.00 ± 0.00) punctae also had 35S:GFP–RABA1c. The strong to moderate localization of FL1, FL3, and FL7 proteins to a RABA1c-labelled compartment suggests that, like FKD1, these proteins may be acting in the post-Golgi secretory pathway.

### Mutations to *FL* gene family members enhance *fkd1* leaf vein pattern phenotype

**Fig. 6.**
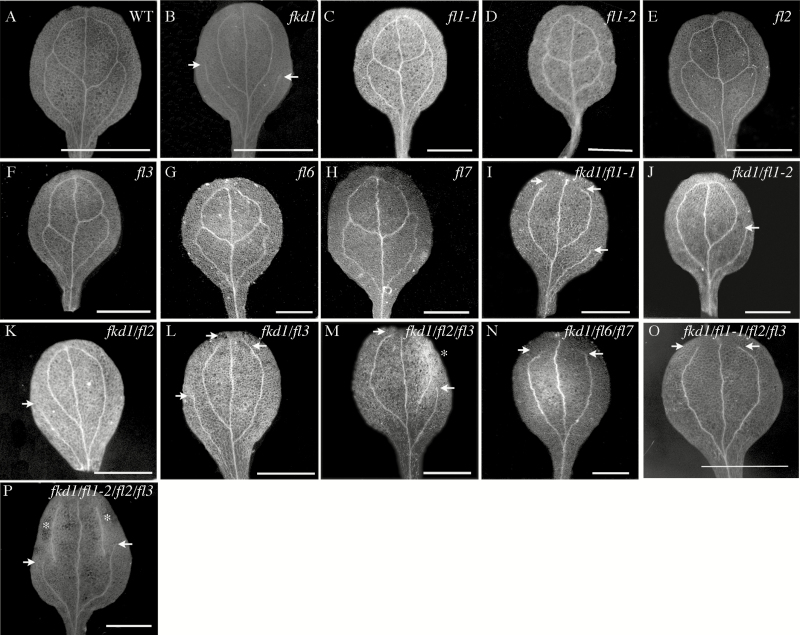
Vascular patterns of cotyledons of various genotypes at 14 DAG. (A) Wild type; (B) *fkd1*; (C) *fl1-1*; (D) *fl1-2*; (E) *fl2*; (F) *fl3*; (G) *fl6*; (H) *fl7*; (I) *fkd1*/*fl1-1*; (J) *fkd1*/*fl1-2*; (K) *fkd1*/*fl2*; (L) *fkd1*/*fl3*; (M) *fkd1*/*fl2/fl3*; (N) *fkd1*/*fl6/fl7*; (O) *fkd1*/*fl1-1/fl2/fl3*; and (P) *fkd1*/*fl1-2/fl2/fl3*. Arrows indicate non-meeting secondary veins, and asterisks indicate VIs (scale bar=2 mm).

**Fig. 7.**
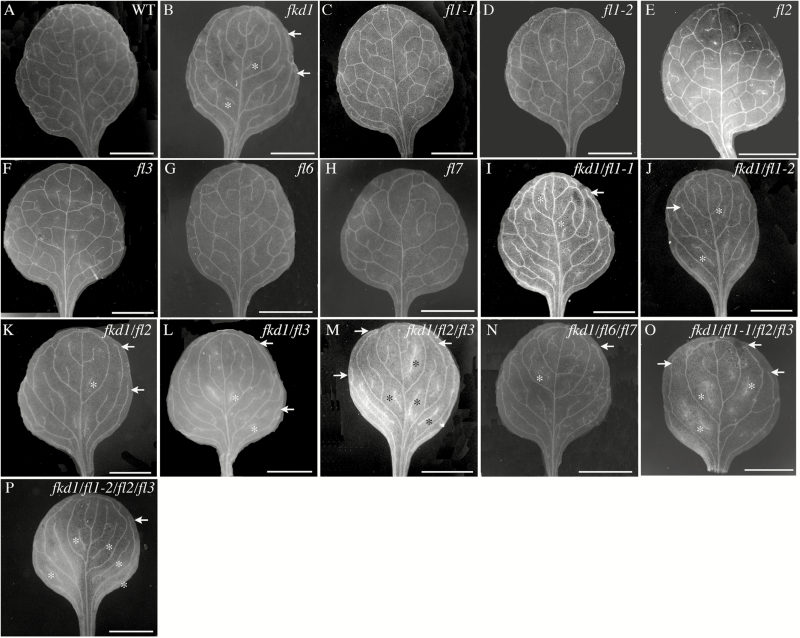
Vascular patterns of first leaves of different genotypes at 21 DAG. (A) Wild type; (B) *fkd1*; (C) *fl1-1*; (D) *fl1-2*; (E) *fl2*; (F) *fl3*; (G) *fl6*; (H) *fl7*; (I) *fkd1*/*fl1-1*; (J) *fkd1*/*fl1-2*; (K) *fkd1*/*fl2*; (L) *fkd1*/*fl3*; (M) *fkd1*/*fl2/fl3*; (N) *fkd1*/*fl6/fl7*; (O) *fkd1*/*fl1-1/fl2/fl3*; and (P) *fkd1*/*fl1-2/fl2/fl3*. Arrows indicate non-meeting secondary veins, and asterisks indicate VIs (scale bar=2 mm).

Mutation to the *FKD1* gene results in cotyledons and leaves with open venation due to lack of distal junctions between secondary and tertiary veins (Steynen and Schultz, 2003). The sequence similarity and partial co-localization of members of the FL family with FKD1 suggest that they might play a redundant role with FKD1 in vein pattern formation. To test this idea, we identified insertion mutants in three group 1 family members (*FL1*, *FL2*, and *FL3*) and two group 3 family members (*FL6* and *FL7*) by PCR using primers specific for the genes and the T-DNA (Supplementary Table S2). The position of the T-DNA in each allele was confirmed by sequencing PCR products (Supplementary Fig. S1A), and alleles were confirmed to be null (*fl1-2*, *fl2*, *fl3*, *fl6*, and *fl7*) or partial loss of function (*fl1-1*) by RT-PCR (Supplementary Fig. 1B). No obvious defects in cotyledon or first leaf vascular patterning are seen in homozygous single mutant lines (Figs 6C–H, 7C–H), although some changes in leaf area and vein density are statistically significant (Tables 2, 3). To test for functional redundancy with *FKD1*, we generated double, triple, and quadruple mutants of *fkd1* with other *FL* gene family insertion lines, and cotyledons and leaves of mutant lines were analysed for area, vein density, and pattern defects, including non-meeting secondary veins and VIs (Tables 2, 3; Figs 6, 7).

Further reduction of group 1 gene function by introducing mutations within group 1 genes (*fl1-1*, *fl1-2*, *fl2*, and *fl3*) into *fkd1* results in progressively increased frequency of non-meeting veins, as indicated by increased numbers of non-meeting secondary veins and increased numbers of VIs in both cotyledons and first leaves compared with *fkd1* (Tables 2, 3; Figs 6, 7). Double mutants between *fkd1* and *fl1-1* (Fig. 7I) or *fl3* (Fig. 7L) result in a higher number of non-meeting secondary veins in first leaves compared with *fkd1* (Fig. 7B). *fkd1*/*fl1*-2 (Fig. 6J), *fkd1/fl2* (Fig. 6K), and *fkd1/fl3* (Fig. 6L) double mutants have more VIs in cotyledons and *fkd1*/*fl1*-2 (Fig. 7J) and *fkd1/fl2* (Fig. 7K) also have more VIs in first leaves. First leaves of *fkd1/fl2/fl3* triple (Fig. 7M) and *fkd1/fl1-1/fl2/fl3* (Fig. 7O) and *fkd1/fl1-2/fl2/fl3* (Fig. 7P) quadruple mutants have more VIs than any double mutant and, compared with the double mutants, the cotyledons have fewer secondary veins that are almost entirely non-meeting (Fig. 6M, O, P). In these mutants, the smaller area and presence of VIs (which, because they are disconnected cannot be assigned a hierarchy level) results in fewer secondary veins compared with *fkd1*. These results suggest that the other group 1 genes act redundantly with *FKD1* to allow vein meeting and maintain vein continuity. In contrast, introduction of group 3 mutations (*fl6* and *fl7*) into the *fkd1* genotype has little effect on vein meeting, except in cotyledons where the number of non-meeting secondary veins is slightly reduced (Table 2; Fig. 6N).

**Table 2.** Area and vein characteristics of 14 DAG cotyledons from various genotypes

Genotype	Sample size	Area (mm^2^)	Vein density mm/mm^2^	Total secondary veins	Non-meeting secondary veins	Vascular islands
WT	83	6.5 ± 1.5	1.6 ± 0.3	3.8 ± 0.4	0.7 ± 0.8	0
*fkd1*	84	4.6 ± 1.8^a^	1.9 ± 0.4^a^	3.2 ± 0.7^a^	2.6 ± 0.8^a^	0
*fl1-1*	23	7.3 ± 1.2^a,b^	1.6 ± 0.1^b^	3.8 ± 0.4^b^	1.0 ± 0.8^b^	0
*fl1-2*	15	5.6 ± 2.7	1.9 ± 0.5^a^	4.0 ± 0^b^	0 ± 0^a,b^	0
*fl2*	54	6.6 ± 1.3^b^	**1.7 ± 0.3^b^**	3.7 ± 0.5^b^	0.9 ± 0.7^b^	0
*fl3*	45	6.9 ± 2.2^b^	1.7 ± 0.2^a,b^	3.7 ± 0.5^b^	0.7 ± 0.8^b^	0
*fkd/fl1-1*	24	**3.4 ± 1.2^a,b,c^**	2.3 ± 0.4^a,b,c^	2.9 ± 0.7^a,c^	2.6 ± 0.8^a,c^	0
*fkd1/fl1-2*	22	6.3 ± 0.9^b^	1.5 ± 0.1^b,c^	3.1 ± 0.8^a,c^	2.1 ± 1.3^a,b,c^	1.0 ± 0.0^a,b,c^
*fkd1/fl2*	41	5.3 ± 1.4^a,b,c^	2.0 ± 0.3^a,c^	3.4 ± 0.6^a,c^	2.4 ± 1.1^a,c^	**1.0 ± 0.0^a,b,c^**
*fkd1/fl3*	58	6.9 ± 1.60^b^	1.6 ± 0.26^b,c^	2.7 ± 0.7^a,b,c^	1.9 ± 0.9^a,b,c^	1.0 ± 0.0^a,b,c^
*fkd1/fl2/fl3*	71	6.8 ± 1.9^b,d^*	**1.4 ± 0.3^a,b,c,d^**	2.4 ± 0.5^a,b,c,d^	1.9 ± 0.8^a,b,c,d^*	1.5 ± 0.7^a,b,c,d^
*fkd1/fl1-1/fl2/fl3*	67	4.5 ± 1.9^a,c,d,e^	1.8 ± 0.4^a,c^*^,d,e^	2.6 ± 0.7^a,b,c,d^*	2.3 ± 0.9^a,b,c,d^*^,e^	0
*fkd1/fl1-2/fl2/fl3*	19	6.8 ± 1.1^b,d^*	**1.4 ± 0.1^a,b,c,d^**	2.7 ± 0.8^a,b,c,d^*^,e^	2.6 ± 0.8^a,c,d^*^,e^	1.2 ± 0.6^a,b,c,d^
*fl6*	38	6.7 ± 1.9^b^	1.8 ± 0.7^a^	3.7 ± 0.6^b^	1.8 ± 1.6^a,b^	0
*fl7*	21	7.4 ± 1.5^a,b^	1.6 ± 0.3^b^	3.9 ± 0.5^b^	1.0 ± 0.8^b^	0
*fkd1/fl6/fl7*	25	4.6 ± 1.0^a,c^	1.8 ± 0.2^a,c^*	3.2 ± 0.8^a,c^	2.1 ± 1.0^a,b,c^*	0

All values are means ±SD. ^a^ is significantly different from the wild type (bold), ^b^ is significantly different from *fkd1* (mutant combinations with *fkd1* that are statistically different from *fkd1* are underlined), ^c^ is significantly different from single mutants, ^d^ is different from double mutants, and ^e^ is different from the triple mutant. * represents a significant difference from one of the tested double mutants, but not all.

**Table 3.** Area and vein characteristics of 21 DAG first leaves from various genotypes

Genotype	Sample size	Leaf area mm^2^	Leaf vein density mm mm^–2^	Number of secondary veins	Non-meeting secondary veins	Vascular islands
WT	134	34.3 ± 9.0	2.0 ± 0.2	7.7 ± 1.0	0.7 ± 0.8	0
*fkd1*	94	**39.3 ± 14.1^a^**	**1.7 ± 0.2^a^**	**7.1 ± 1.2^a^**	**3.6 ± 1.4^a^**	**1.2 ± 1.0^a^**
*fl1-1*	23	**38.3 ± 6.7^a^**	2.0 ± 0.1^b^	7.7 ± 0.6^b^	**0.2 ± 0.5^a,b^**	0
*fl1-2*	15	31.8 ± 10.3	1.9 ± 0.2^b^	**6.8 ± 1.1^a^**	0.5 ± 0.6^b^	0
*fl2*	57	36.7 ± 11.6	**1.9 ± 0.4^a,b^**	7.6 ± 0.7^b^	0.7 ± 0.9^b^	0
*fl3*	97	42.5 ± 11.1^a^	**1.7 ± 0.2^a^**	7.5 ± 1.0^b^	**0.2 ± 0.5^a,b^**	0
*fkd1/fl1-1*	27	**30.3 ± 7.8^a,b,c^**	**1.9 ± 0.1^a,b,c^**	7.3 ± 1.5	**4.6 ± 1.5^a,b,c^**	**1.5 ± 1.3^a,c^**
*fkd1/fl1-2*	24	**20.1 ± 5.6^a,b,c^**	**2.5 ± 0.3^a,b,c^**	**6.8 ± 0.8^a^**	**3.6 ± 1.4^a,c^**	**2.5 ± 0.9^a,b,c^**
*fkd1/fl2*	35	**30.4 ± 6.2^a,b,c^**	**1.9 ± 0.1^a,b^**	**6.4 ± 1.4^a,b,c^**	**3.9 ± 1.2^a,c^**	**2.4 ± 1.4^a,b,c^**
*fkd1/fl3*	46	36.2 ± 9.8^c^	**1.9 ± 0.1^a,b,c^**	7.6 ± 0.9^b^	**4.4 ± 1.1^a,b,c^**	**1.1 ± 0.4^a,c^**
*fkd1/fl2/fl3*	96	**25.5 ± 9.4^a,b,c,d^**	2.0 ± 0.3^b,c^*^,d^*	**5.0 ± 1.3^a,b,c,d^**	**2.8 ± 1.1^a,b,c,d^**	**4.9 ± 2.2^a,b,c,d^**
*fkd1/fl1-1/fl2/fl3*	69	**17.5 ± 5.7^a,b,c,d,e^**	**2.3 ± 0.30^a,b,c,d,e^**	**3.8 ± 1.2^a,b,c,d,e^**	**1.6 ± 1.1^a,b,c,d,e^**	**3.7 ± 1.3^a,b,c,d^**
*fkd1/fl1-2/fl2/fl3*	96	**23.9 ± 6.05^a,b,c,d^**	**2.1 ± 0.2^a,b,c,d^**	**4.2 ± 1.3^a,b,c,d,e^**	**2.07 ± 1.32^a,b,c,d,e^**	**4.7 ± 2.1^a,b,c,d^**
*fl6*	32	**29.5 ± 8.5^a,b^**	**1.7 ± 0.2^a^**	**6.3 ± 1.3^a,b^**	**0.4 ± 0.5^a,b^**	0
*fl7*	30	**27.6 ± 6.2^a,b^**	2.0 ± 0.3^b^	7.3 ± 1.1	0.6 ± 0.7^b^	0
*fkd1/fl6/fl7*	31	**30.7 ± 6.70^a,b^**	2.0 ± 0.2^b,c^*	**6.9 ± 1.4^a^**	**3.09 ± 1.33^a,c^**	**1.7 ± 1.3^a,c^**

All values are means ±SD. ^a^ is significantly different from the wild type (bold), ^b^ is significantly different from *fkd1* (mutant combinations with *fkd1* that are statistically different from *fkd1* are underlined), ^c^ is significantly different from single mutants, ^d^ is different from double mutants, and ^e^ is different from the triple mutant. * represents a significant difference from one of the tested double mutants, but not all.

The effects of mutations of *FL* genes on leaf and cotyledon area and vein density are quite varied (Tables 2, 3; Figs 6, 7). Leaf area and vein density tend to be negatively correlated (Sack *et al.*, 2012). Group1 (*fkd1*, *fl1-1*, and *fl3*) single mutants have larger leaves than the wild type, with a corresponding reduction in vein density, whereas group 3 (*fl6* and *fl7*) mutants have smaller leaves with either no change in density (*fl7*) or a reduction in vein density (*fl6*). Surprisingly, all triple and quadruple mutants have smaller leaves than the wild type but, except for *fkd1/fl1-1/fl2/fl3*, do not show the expected increase in vein density. These phenotypes suggest that the *FL* gene family may co-ordinate leaf size with vein density and, further, that the group 1 members and group 3 members may have opposing functions.

### PIN1–GFP expression and localization is defective in group 1 triple mutants

PIN1 expression is the earliest known determinant for leaf vein formation in Arabidopsis (Scarpella *et al.*, 2006; Wenzel *et al.*, 2007). Since the vein non-meeting in *fkd1* leaves has been correlated with defective PIN1 localization during vein development (Hou *et al.*, 2010), we introduced PIN1–GFP into the *fkd1/fl2/fl3* triple mutant line to determine if the increased number of non-meeting veins of the group 1 multiple mutants might result from more extreme defects to PIN1 localization.

PIN1 expression and localization during leaf development have been described previously (Scarpella *et al.*, 2006; Wenzel *et al.*, 2007), so we will only describe that associated with secondary vein formation. The early second-order PEDs, referred to as the lower loop domain (LLD), emerge in association with auxin convergence points along the leaf margin and are comprised of a transient wide distal section near the marginal convergence point and a persistent narrow proximal section near the midvein. PIN1 localization is lateral and basal in the distal section and basal in the proximal section. After the LLD is fully connected to the midvein PED, initiation of the upper loop domain (ULD) occurs. The ULD gradually extends from the LLD toward the distal midvein to become a ‘connected’ PED. Once the ULD connects to the midvein, the transient wide section of the LLD section near the marginal convergence point disappears. Connected ULDs comprise two segments, one at the midvein where PIN1 localization is apical and one at the LLD where PIN1 localization is basal. The two segments of opposite polarity are bridged by a single cell (Scarpella *et al.*, 2006).

**Fig. 8.**
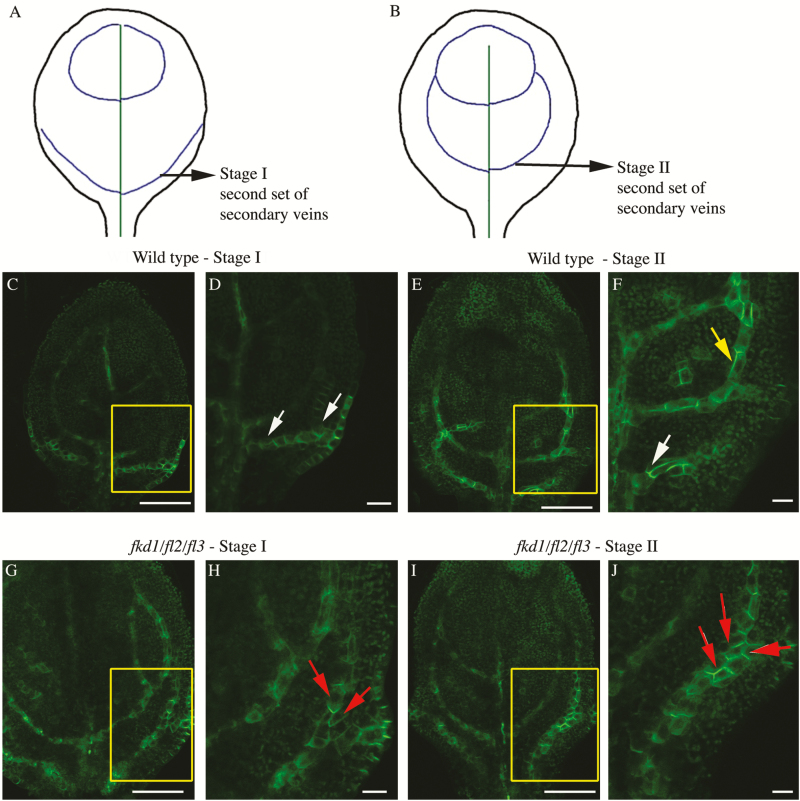
PIN1–GFP expression in the developing second set of secondary veins in the wild type and *fkd1/fl/2/fl3* triple mutant. (A and B) Diagram of successive stages of development of the second set of secondary veins; lines indicate PIN–GFP expression domains (PEDs). (C–F) PIN1–GFP expression in the second set of secondary veins of wild-type stage I (C, D) and stage II (E, F) first leaf (boxed area in C and E enlarged in D and F, respectively). (G–J) PIN1–GFP expression in the second set of secondary veins of the *fkd1/fl2/fl3* triple mutant stage I (G, H) and stage II (I, J) first leaf (boxed area in G and I enlarged in H and J, respectively). White arrows indicate basal PIN1–GFP, yellow arrows indicate lateral PIN1–GFP, and red arrows indicate PIN1 localization on all sides (scale bar=10 μm).

**Fig. 9.**
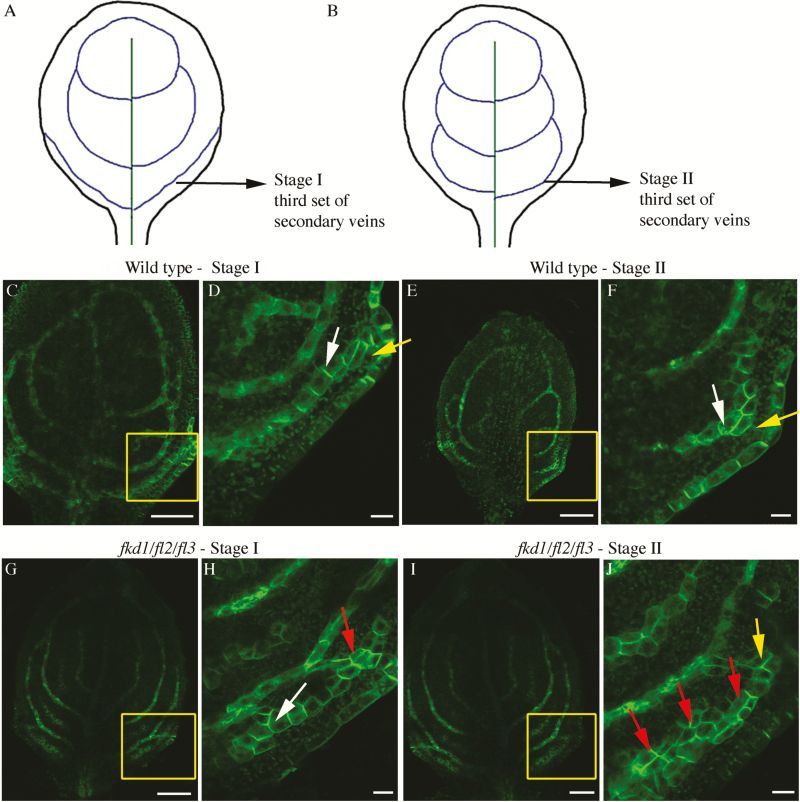
PIN1–GFP expression in the developing third set of secondary veins in the wild type and *fkd1/fl2/fl3* triple mutant. (A and B) Diagram of successive stages of development of the third set of secondary veins; lines indicate PIN–GFP expression domains (PEDs). (C–F) PIN1–GFP expression in the third set of secondary veins of the wild-type stage I (C, D) and stage II (E, F) first leaf (boxed area in C and E enlarged in D and F, respectively). (G–J) PIN1–GFP expression in the third set of secondary veins of the *fkd1/fl2/fl3* triple mutant stage I (G, H) and stage II (I, J) first leaf (boxed area in G and I enlarged in H and J, respectively). White arrows indicate basal PIN1–GFP, yellow arrows indicate lateral PIN1–GFP, and red arrows indicate PIN1 localization on all sides (scale bar=10 μm).

We focused on comparing PIN1–GFP expression and localization within the second and third set of secondary veins between the wild type and *fkd1/fl2/fl3* triple mutants (Figs 8, 9). While these veins always meet the midvein distally in wild-type leaves (*n*=68), only 18% of the second set and 32% of the third set meet distally in *fkd1/fl2/fl3* (*n*=95) (Table 4). Because leaf formation is slightly delayed in *fkd1/fl2/fl3*, DAG was not a reliable measure for comparison. Instead, we compared leaves at the same stage of vein development: stage I refers to the stage of secondary vein formation that includes the transient wide PED adjacent to the margin (Figs 8A, C, D, 9A, C, D); Stage II refers to the stage of development in which the transient wide PED is absent and the ULD of the secondary vein has formed (Figs 8B, E, F, 9B, E, F).

**Table 4.** Comparison of secondary vein characteristics (second and third secondary veins) between the wild type (WT) and the *fkd1/fl2/fl3* triple mutant

A. Genotype (*n*=68 for the WT and *n*=95 for *fkd1/fl2/fl3*)	Number of non-meeting secondary veins		Percentage of non-meeting secondary veins	
WT second secondary vein	0		0	
*fkd1/fl2/fl3* second secondary vein	36*		18.9	
WT third secondary vein	0		0	
*fkd1/fl2/fl3* third secondary vein	60 *		31.6	
B. Genotype (*n*=45 for the WT and *n*=65 for *fkd1/fl2/fl3*)	Percentage wide PED			
WT second secondary vein stage I	10.7			
*fkd1/fl2/fl3* second secondary vein stage I	30.3*			
WT second secondary vein stage II	1.1			
*fkd1/fl2/fl3* second secondary vein stage II	16.0*			
WT third secondary vein stage I	26.4			
*fkd1/fl2/fl3* third secondary vein stage I	28.2			
WT third secondary vein stage II	10.0			
*fkd1/fl2/fl3* third secondary vein Stage II	25.2			
C. Genotype (*n*=15 for both genotypes)	Percentage PIN1–GFP localization			
	Apical	Basal	Lateral	All sides
WT second secondary vein stage I	7.8	40.8	51.3	0
*fkd1/fl2/fl3* second secondary vein stage I	2.8	25.0	25.0	47.2*
WT second secondary vein stage II	43.3	37.8	18.9	0
*fkd1/fl2/fl3* second secondary vein stage II	13.0*	19.3*	14*	53.3*
WT third secondary vein stage I	3.0	56.7	40.0	0
*fkd1/fl2/fl3* third secondary vein stage I	5.2	26.5*	15.4*	52.9*
WT third secondary vein stage II	40.9	40.9	18.1	0
*fkd1/fl2/fl3* third secondary vein stage II	15.0*	18.3*	19.0	47.7*

(A) Frequency of non-meeting secondary veins, analysed at 21 DAG; (B) wide (three or more cells) PIN–GFP expression domains (PEDs) at stage I and stage II of development; (C) localization of PIN1–GFP within PED cells to different membrane faces at stage I and stage II of development.

*Significant difference (*P*<0.05) from the wild type; where percentage values are shown, significant differences were calculated from raw data using Fisher’s test.

Generally, PEDs in *fkd1/fl2/fl3* secondary veins are wider than in the wild type. Comparing stage I of the second secondary veins, only 11% of wild-type PEDs are three or more cells wide (Fig. 8C, C; Table 4), whereas 30% of *fkd1/fl2/fl3* PEDs are three or more cells wide (Fig. 8G, H; Table 4). At stage II, wild-type second secondary vein PEDs are mostly restricted to a single cell file with only 1% more than three cells wide (Fig. 8E, F), whereas in *fkd1/fl2/fl3*, restriction of the PED into a single cell file occurs only in 4% of secondary veins, and 16% of PEDs remain wider than three cells (Fig. 8I, J; Table 4). Although not statistically significant (Table 4), a higher percentage (25%) of the third secondary veins remains wide in stage II of *fkd1/fl2/fl3* (Fig. 9J) compared with the wild type (10%, Fig. 9H).

### Group 1 *FL* gene family mutations affect shoot and root development

To determine whether mutations in group 1 *FL* gene family members affect auxin transport-related developmental processes other than leaf vein pattern, phenotypes including flowering time, number of rosette leaves and bracts, root length, and gravitropic response were compared between the wild type, *fkd1*, the *fkd1/fl2/fl3* triple mutant, and the *fkd1/fl1-2/fl2/fl3* quadruple mutant (Table 5).

**Table 5.** Comparison of shoot and root characteristics between the wild type (WT), *fkd1*, the *fkd1/fl2/fl3* triple mutant, and the *fkd1/fl1-2/fl2/fl3* quadruple mutant

Genotype	Days to flower	Rosette leaves	Bracts	Root growth over 24 h	Root curvature after 6 h
WT	19 (16)	6.4 ± 1.0 (16)	2.2 ± 0.4 (16)	3.9 ± 1.1 (19)	81.0 ± 15.2 (19)
*fkd1*	19 (15)	6.4 ± 0.9 (15)	2.2 ± 0.4 (15)	3.1 ± 1.4^a^ (25)	81.6 ± 17.8 (21)
*fkd1/fl2/fl3*	23 (16)	6.5 ± 1.1 (16)	2.6 ± 0.5 (16)	2.5 ± 0.9^a^ (21)	76.6 ± 13.8^a^ (25)
*fkd1/fl1-2/fl2/fl3*	27 (16)	8.9 ± 0.8^a,b,c^ (16)	2.9 ± 0.8 (16)	1.4 ± 0.8^a,b,c^ (18)	60.6 ± 27.5^a,b,c^ (18)

Days to flower was defined as the time at which half the population had an open flower. Number of rosette leaves and bracts was counted at that time. Root growth was measured over 24 h in seedlings from 4 to 5 DAG, and root curvature of 5 DAG seedlings was measured 6 h after a 90° rotation. Numbers in parentheses are the sample size.

^a^ represents a significant difference from the wild type, ^b^ represents a significant difference from *fkd1*, and ^c^ represents a significant difference from the triple mutant (*P*<0.05).

*fkd1/fl1-2/fl2/fl3* quadruple mutants produce significantly more rosette leaves and flower later than wild-type, *fkd1*, and *fkd1/fl2/fl3* triple mutant lines (Table 5; Supplementary Fig. S2). Compared with the wild type and *fkd1*, *fkd1/fl2/fl3* and *fkd1/fl1-2/fl2/fl3* roots grow significantly less over a 24 h period (Table 5). Six hours following a 90° rotation, both *fkd1/fl2/fl3* and *fkd1/fl1-2/fl2/fl3* show reduced root tip curvature compared with the wild type and *fkd1*, with *fkd1/fl1-2/fl2/fl3* being more extreme than *fkd1/fl2/fl3* (Table 5).

The reduced root growth and gravitropic response of the multiple mutants suggests that auxin transport may be defective in these lines; however, we observed no difference in PIN1 localization in root vascular cells of the wild type compared with *fkd1/fl2/fl3* (Supplementary Fig. S3A, D). To determine if, like *sfc* mutations (Sieburth *et al.*, 2006), the *FL* mutations caused increased brefeldin A (BFA) sensitivity in roots, BFA wash out experiments were performed in the wild type and *fkd1/fl2/fl3* expressing PIN1–GFP. In both wild-type and *fkd1/fl2/fl3* root cells, PIN1–GFP accumulates in BFA compartments after treatment with BFA (Supplementary Fig. S3B, E). After BFA wash out, the intracellular aggregates decrease and the plasma membrane signals are recovered in both genotypes (Supplementary Fig. S3C, F), suggesting that the root defects are not correlated with enhanced BFA sensitivity.

## Discussion

### Group 1 of the *FL* gene family acts redundantly to localize PIN1 in provascular cells

Analysis of lines mutant for group 1 genes within the *FL* gene family indicates that these genes act redundantly with *FKD1* to control vein connectivity and vein density in cotyledons and leaves. The number of secondary veins that fail to connect distally, and the number of disconnected veins (VI) increases as other group 1 genes (*FL1*, *FL2*, and *FL3*) are mutated within the *fkd1* genotype. Consistent with redundant action, all four group 1 *FL* genes are expressed within developing leaves (http://travadb.org; Klepikova *et al.*, 2016). The non-meeting veins in *fkd1* mutants are correlated with subtle defects to PIN1–GFP localization early in vein development and, in particular, with an absence of apically localized PIN1–GFP (Hou *et al.*, 2010). In developing secondary veins of the *fkd1/fl2/fl3* triple mutant, mislocalization of PIN1–GFP is severely compromised: compared with the wild type, PIN1–GFP is less frequently localized to either apical or basal membrane faces, but is frequently symmetrically localized to all cell faces. The early mislocalization of PIN1–GFP is correlated with a high proportion of non-meeting secondary veins and VIs, suggesting that the vein discontinuities seen in the group 1 mutant lines are the result of defects to PIN1 localization, and that these genes act redundantly with *FKD1* to localize PIN1. The short roots and lack of gravitropic response within *fkd1/fl2/fl3* triple and *fkd1/fl1-2/fl2/fl3* quadruple mutants suggest that auxin transport within roots may also be defective. While we could detect no defects to PIN1–GFP within the triple mutant roots in either the presence or absence of BFA, it is possible that mislocalization of other PIN proteins accounts for the root phenotypic defects.

During vein formation, dynamic relocalization of PIN proteins is integral to the canalization process, which allows narrowing of PIN1 expression domains (Scarpella *et al.*, 2006; Wenzel *et al.*, 2007; Bayer *et al.*, 2009). Defects to PIN1 localization in *fkd1* single mutants are correlated with wider PEDs (Hou *et al.*, 2010), and this effect is more extreme in *fkd1/fl2/fl3* triple mutant leaves. For example, the PED remained wide (three or more cells) in only 1% of wild-type but 16% of *fkd1/fl2/fl3* triple mutant secondary veins; at a similar stage, 7% of *fkd1* secondary vein PEDs remain wide (Hou *et al.*, 2010). The failure of *fkd1/fl2/fl3* triple mutants to canalize PIN1–GFP expression, correlated with more extreme defects to PIN–GFP localization, highlights the importance of directed auxin transport to auxin canalization. We have previously shown that *FKD1* is transcriptionally activated by auxin, and proposed that it forms part of the autoregulatory loop that links PIN1 localization to auxin canalization, which subsequently influences auxin-induced gene expression (Hou *et al.*, 2010). Interestingly, like *FKD1*, all of the other members within the group 1 *FL* gene family have predicted upstream ARF-binding sequences (data not shown), suggesting that they may be part of the same autoregulatory loop.

### *FL* gene family mutations affect leaf size and vein density

The trend towards smaller leaves having higher vein density is well conserved throughout angiosperms (Sack *et al.*, 2012), but the mechanism controlling the relationship is not known. Introduction of mutations within group 3 genes (*fl6* and *fl7*) into the *fkd1* background does not increase the severity of non-meeting veins, suggesting that this group is not required for vein meeting. This is especially surprising for FL7, which co-localizes strongly with FKD1–GFP and 35S:GFP–RABA1c, suggesting that FL7 might act with FKD1 in the secretory pathway. One explanation is that *FL7* is only weakly expressed in developing leaves (Klepikova *et al.*, 2016). *fkd1/fl6*/*fl7* triple mutants have leaves that are smaller than the wild type, but maintain the same vein density as the wild type. The formation of smaller leaves with similar vein density to the wild type is also seen in several double, triple, and quadruple mutants within group 1. Our data suggest that group 1 and group 3 genes may act to co-ordinate leaf size with vein density. One possibility is that, as proposed for the *unh* mutant (Pahari *et al.*, 2014), the discontinuous vein pattern results in altered flux in major veins, which in turn affects auxin levels in the leaf lamina. Changing auxin in the lamina is expected to affect both initiation of higher vein orders (Aloni *et al.*, 2003) and cell division and expansion within the leaf (Zgurski *et al.*, 2005), potentially influencing both vein density and leaf size.

### The FL family is localized through the Golgi and post-Golgi system

The severe vein pattern defects of the *fkd1/fl1-2/fl2/fl3* quadruple and *fkd1/fl2/fl3* triple mutants and severe defects to PIN1–GFP localization in *fkd1/fl2/fl3* triple mutants indicate that these genes act redundantly to localize PIN1. Our finding that FKD1 localizes to the TGN, plasma membrane, and to RABA-positive compartments led to the proposal that FKD1 is involved in the post-Golgi secretory pathway (Prabhakaran Mariyamma *et al.*, 2017). Whereas group 1 proteins tested (FL1 and FL3) localize with FKD1 to some extent, the co-localization is incomplete, suggesting that these proteins have a function distinct from FKD1. Interestingly, FL1 and FL3 co-localize more strongly with SYP61 than FKD1 does, suggesting stronger localization to the TGN. Two of the group 3 proteins (FL5 and FL6) localize strongly to the Golgi, based on co-localization with ST–RFP, whereas the third (FL7) localizes weakly to both the TGN and the Golgi. Although FL7 co-localizes strongly with FKD1, mutation to *FL7* and *FL6* in an *fkd1* background has little effect on the *fkd1* phenotype, indicating that the genes do not act redundantly.

The localization of FL proteins to the Golgi (FL5 and FL6), TGN (FL1 and FL3), and RABA-labelled vesicles (FL1, FL3, and FL7) is consistent with members of the family acting, like FKD1, in the secretory pathway. The mislocalization of PIN1–GFP in *fkd1/fl2/fl3* triple mutants suggests that one cargo of these genes is PIN1; the root phenotype but absence of PIN1 localization defects indicates that other cargos exist. FKD1 is strongly associated with SFC (Naramoto *et al.*, 2009), and also with ARF-GTPases of the ARF1 family (Prabhakaran Mariyamma *et al.*, 2017). It will be interesting to assess whether other members of the family associate with ARF-GTPases and ARF-GAPs that localize to the Golgi such as AGD7, which has been proposed to have a role in anterograde trafficking (Min *et al.*, 2007) or to the TGN such as AGD5, which has been proposed to have a role in trafficking to the vacuole (Liljegren *et al.*, 2009; Stefano *et al.*, 2010).

### Evolution of the *FL* gene family

The *FL* gene family encodes proteins with DUF828 and PH or PH_2 domains. The domain combination is unique to the plant kingdom, and its expansion can be correlated with key events in plant kingdom evolution. The presence of a single DUF828-containing gene within the genome of the liverwort *M. polymorpha* and the moss *P. patens* indicates coincident origin of the family with the emergence of terrestrial plants ~443–490 million years ago (Douzery *et al.*, 2004), pre-dating the origin of vascular tissue. Polar targeting of PIN proteins and PIN protein-mediated auxin transport is responsible for gravitropic responses, and gametophyte, sporophyte, and leaf development in *P. patens* (Bennett *et al.*, 2014), implying a key role for PIN protein localization in the early evolution of plant form. In both gymnosperms (*P. abies*) and *A. trichopoda*, a sister species to all other extant angiosperms (Chamala *et al.*, 2013), the four *FL* gene copies are separated into two well-supported clades, suggesting at least two gene copies existed in the common ancestor. Interestingly, in the basal angiosperm *A. trichopoda*, the emergence of a reticulate vein pattern (Takhtajan, 2009) is correlated with the emergence of a single gene that falls within group 1, which also contains *FKD1*, *FL1*, *FL2*, and *FL3.* Since the absence of these genes in Arabidopsis results in failure to form a reticulate vein pattern, we propose that the emergence of the gene in *Amborella* may have been important for the formation of the reticulate vein pattern characteristic of angiosperms.

Vein traits contribute to greater performance of plants, and angiosperms evolved distinctive vein traits compared with their earlier evolved lineages. Early angiosperms possessed lower order veins with less organization, whereas more derived angiosperms had increasing numbers of vein orders (Brodribb and Feild, 2010; Sack and Scoffoni, 2013) with the hierarchy of vein orders forming the reticulate mesh typical of angiosperms (McKown *et al.*, 2010). Subsequently, larger leaves with large major veins for mechanical support and a high leaf vein length per unit area (VLA) enabled better transpirational cooling and higher photosynthetic rates (Sack *et al.*, 2012). Multiple mutations in group 1 result in leaves lacking the reticulate mesh, and having a low vein density despite their small size. It is possible that the expansion of the group 1 *FL* genes within angiosperms enabled the formation of high vein density and improved vein connections, characteristics proposed to contribute to the success of angiosperms.

## Supplementary data

Supplementary data are available at *JXB* online.

Table S1. Domain organization [DUF828 (IPR00856), Pleckstrin-like domain (PH_2; IPR013666), and Pleckstrin homology domain (PH; IPR001849)] within Arabidopsis *FL* gene family members.

Table S2. Primers used to identify T-DNA insertions and sequence flanking regions in *FL* gene family alleles or assess gene transcript presence in *FL* gene family alleles by RT-PCR.

Fig. S1. Position of T-DNA insertions and left and right primers used for RT-PCR amplification, and RT-PCR amplification of *FL* and *PP2A* gene transcripts in alleles of *FL* gene family members.

Fig. S2. Adult shoot phenotype of different genotypes at 27 DAG.

Fig. S3. PIN1 trafficking is not altered in *fkd1/fl2/fl3* triple mutant roots.

## Supplementary Material

Supplementary Tables and Figures
