# Supplementary material for: Members of the Arabidopsis FORKED1-LIKE gene family act to localize PIN1 in developing veins
Source: J Exp Bot. 2018 Jul 3;69(20):4773–90. doi: 10.1093/jxb/ery248 (PMC6137986; doi:10.1093/jxb/ery248)
Supplement: Supplementary Tables and Figures [file ery248_suppl_supplementary_tables_figures.pdf]

Supplementary Table 1: Domain organization [DUF828 (IPR00856), Pleckstrin like domain (PH_2; IPR013666) and Pleckstrin Homology domain (PH; IPR001849) within Arabidopsis *FL* gene family members. Numbers indicate amino acid position of domains within the predicted protein, (-) indicates that protein does not contain that domain. Percentage amino acid identity of the FKD1 full-length protein, the FKD1 DUF828 domain and the FKD1 PH_2 domain relative to the corresponding domain region in each member of the gene family.

FL family members	Position of Domains (aa)			Amino acid sequence identity (%)		
	DUF828	PH_2	PH	FKD1	DUF828	PH/PH_2
FKD1 (At3g63300)	30-336	379-483	-			
FL1 (At5g43870)	22-307	341-446	-	53.90	57.70	64.20
FL2 (At3g22810)	18-312	358-462	353-463	56.10	59.40	59.70
FL3 (At4g14740)	19-178	224-328	-	56.10	57.60	68.60
FL4 (At4g32780)	54-263	-	-	27.10	34.20	-
FL5 (At4g17350)	44-86<br>111-258	283-386	278-386	28.80	26.00	39.80
FL6 (At4g16670)	58-93<br>126-174	307-411	302-411	27.30	17.60	28.80
FL7 (At5g47440)	43-108<br>133-257	283-385	278-386	27.1	25.20	34.10
FL8 (At5g57770)	21-268	289-390	-	26.20	20.80	20.60

Supplementary Table 2: Primers used to A) identify T-DNA insertions and sequence flanking regions in *FL* gene family alleles or B) assess gene transcript presence in *FL* gene family alleles by RT-PCR. Primers also used for sequencing are in bold, * indicates that the T-DNA left border primer used in sequencing was CGTCCGCAATGTGTTATTAAG, ** indicates that the T-DNA left border primer used in sequencing was ATTTTGCCGATTTCGGAAC.

allele	Left Primer	Right Primer	Annealing temperature	Number of cycles
**A)** fl1-1	GGCATAAGACTTTAGATGGTAGC	**GCCATGATACGTTCCATGACC***		
fl1-2	GCTCATTCACCGACAGTCCTCCG	**GGCTGTTGAGATAGACCGTTGTG***		
fl2	**CACTGCAACAACTACACAGTCC****	**CGTGAAGGTCCCTCCTACATGC****		
fl3	GTATCACCAAGAACATCTGGTCGGC	**CGTGAATCTGAGCGTTATGAGCCCG***		
fl6	GGCTGCTCAATGTGTGGAAG	GCCAAGAAATGGTTTTAAGCAGA<br>**CTTCTTCCAAGATGTTGCTGC****		
fl7	CAAACAACACACCAACCCAAGC	CTCGCTTATTTCGCTGCAATC<br>**TGGCAACTACTGAAAACGACA***		
T-DNA left border	CGTCCGCAATGTGTTATTAAG*<br>ATTTTGCCGATTTCGGAAC**			
**B)** fl1-1 and fl1-2	TTCTACCGTGTGAGCCCTTC	CAGCCGCAATGTTCCATACC	54ºC	30
fl2	ACA GCG TTA AGA GGA GTG GC	TGA AGA TCA CCT TTG CGG GT	58ºC	35
fl3	ATC TGA GCG TTA TGA GCC CG	AGGCTCTAACACCACCCAAC	58ºC	30
fl6	AGCGAAATAAGCGAGTTTGTGT	CTG AAT CCC AGC AAC CCA TCT	56ºC	30
fl7	GCACGACCGATGAACTCATATCTGC	TTCCGGATGCTATCCACCCATGT	58ºC	35
PP2A	GCAGTATCGCTTCTCGCTCCAGTA	TGTTCTCCACAACCGCTTGGTC	58ºC	30

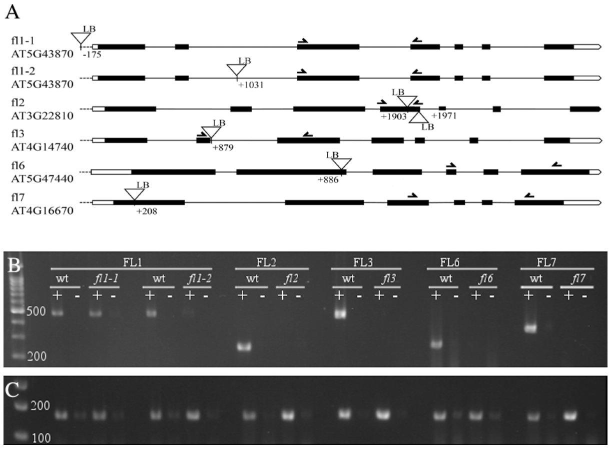

Supplementary Figure 1: Position of T-DNA insertions and Left and Right primers used for RT-PCR amplification (A) and RT-PCR amplification of *FL* (B) and *PP2A* (C) gene transcript in alleles (*fl1-1*, *fl1-2*, *fl2*, *fl3*, *fl6* and *fl7*) of *FL* gene family members. A) For each gene, the transcribed region and area immediately 5' of the transcriptional start-site is drawn. Exons are indicated as boxed regions, with translated sequences shaded and untranslated sequences open. Introns are indicated as lines. Position of left border (LB) is shown on insertion; number adjacent to insertion indicates position of insertion relative to transcription start site. Positions of left (⇀) and right primers (↽) used for RT-PCR are shown on the exons or intron/exon boundaries. (B) RT-PCR using *FL1*, *FL2*, *FL3*, *Fl6* or *FL7* specific primers on RNA transcribed to cDNA with reverse transcriptase (+), or without reverse transcriptase (-) from wild type (wt) or *fl1-1*, *fl1-2*, *fl2*, *fl6* and *fl7* alleles. C) RT-PCR using *PP2A* specific primers from RNA as described in B. 100 and 500 (B) or 100 and 200 (C) bp markers are indicated on ladders to left of each gel.

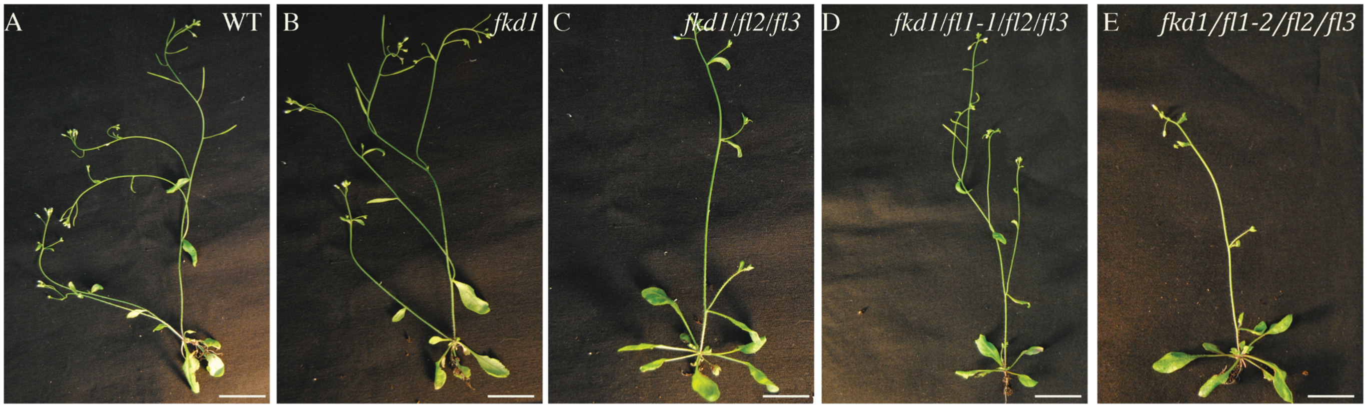

Supplementary Figure 2: Adult shoot phenotype of different genotypes at 27 DAG. (A) Wild type; (B) *fkd1*; (C) *fkd1/fl2/fl3* triple mutant; (D) *fkd1/fl1-1/fl2/fl3* quadruple mutant and (E) *fkd1/fl1-2/fl2/fl3* quadruple mutant (Scale bar = 5mm).

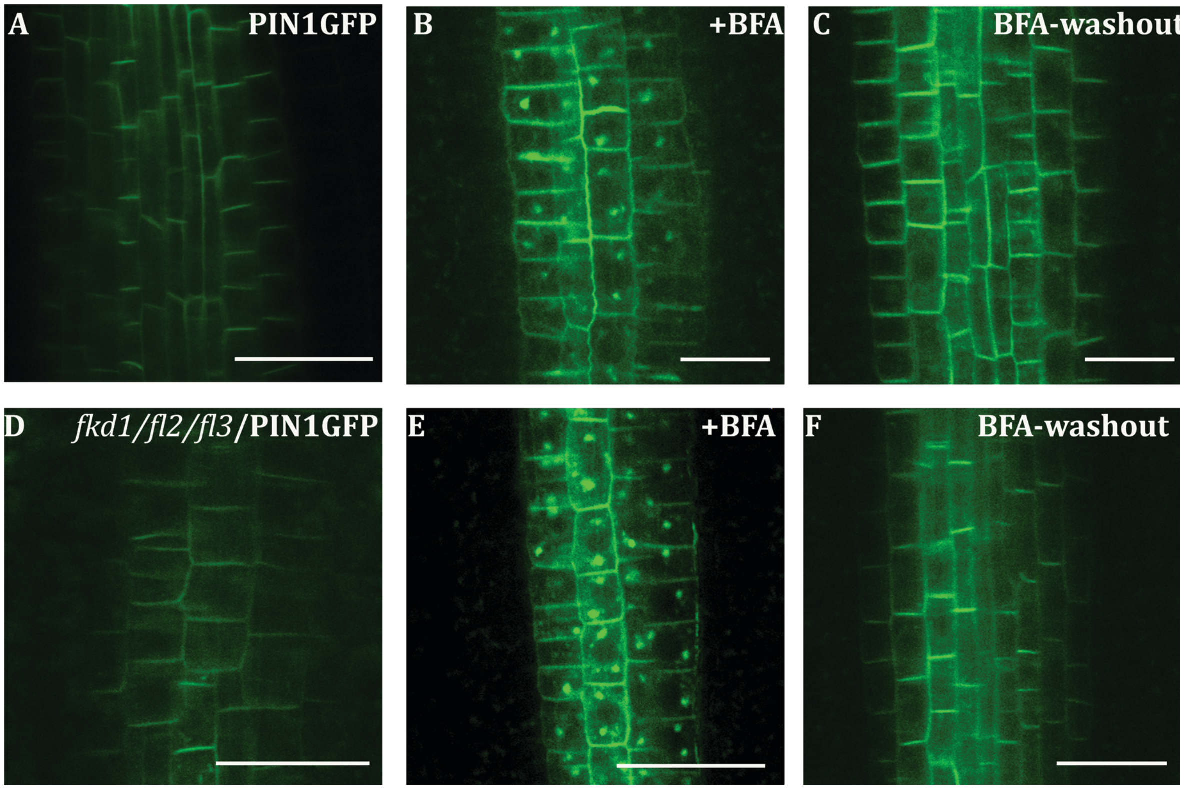

Supplementary Figure 3: PIN1 trafficking is not altered in *fkd1/fl2/fl3* triple mutant roots. Confocal laser scanning microscopy of roots of wild type and *fkd1/fl2/fl3* triple mutants expressing PIN1-GFP at 2.5 DAG, before and after BFA washout (A-F). Polar PM localization of PIN-GFP in root vascular cells of wild type (A-C) and in *fkd1/fl2/fl3* triple mutant (D-F). Localization of PIN1-GFP to plasma membrane of cells in wild type (A) and *fkd1/fl2/fl3* triple mutant (C). Accumulation of PIN1-GFP in BFA compartments in wild type (B) and in *fkd1/fl2/fl3* triple mutant (E). After BFA washout (C and F), PIN1-GFP is observed on PM in wild type (C) and in *fkd1/fl2/fl3* triple mutant (F) to the same extent. Scale bars = 10 µm.
